# ADNP Functions During Early Brain Development and Their Relevance to ASD and ADNP Syndrome

**DOI:** 10.3390/ijms27136085

**Published:** 2026-07-07

**Authors:** Xiaonan Liu, Shiena Watanabe, Sierra Coleman, Vicky Shih, William R. Telfer, Vasu D. Kansagra, Lilit Drak, Laasya Reddy Pesaladinne, Diane Kim, Samridhi Sudan, Anushka Singhal, Kazuhito Toyo-oka

**Affiliations:** Department of Neurobiology and Anatomy, Drexel University College of Medicine, Philadelphia, PA 19129, USA

**Keywords:** ADNP, NAP, early brain development, cortical development, neurogenesis, neurite formation, autism spectrum disorder, ADNP syndrome, microtubule dynamics

## Abstract

The Activity-Dependent Neuroprotective Protein (ADNP) is an important regulator of early brain development, especially during cortical neurogenesis and neurite formation. De novo point mutations or haploinsufficiency of the *ADNP* gene result in ADNP syndrome, which is also known as Helsmoortel-Van der Aa syndrome, a complex neurodevelopmental disorder recognized as a leading single-gene cause of syndromic autism spectrum disorder (ASD) and intellectual disability. ADNP works as both a transcription factor and a microtubule (MT) regulator. As a transcription factor, ADNP is a key component of chromatin remodeling complexes such as ChAHP (CHD4 (Chromodomain Helicase DNA-binding Protein 4)-ADNP-HP1 (Heterochromatin Protein 1)) and SWI/SNF (Switch/Sucrose Non-Fermentable), and it tightly regulates the expression of numerous essential developmental genes. ADNP also modulates the Wnt/β-catenin signaling pathway. During neural differentiation, ADNP is redistributed from the nucleus to the cytoplasm, and this redistribution is regulated by binding to 14-3-3 proteins, which are phosphorylated by protein Kinase C (PKC). After relocating to the cytoplasm, ADNP functions as an MT regulator by binding to microtubule end-binding proteins (EB1 and EB3) and Tau to control neurite formation. Previous studies have focused on NAP (also known as Davunetide, a peptide derived from ADNP) in MT regulation and its therapeutic potential for autism spectrum disorder (ASD) and neurodegenerative diseases, such as Alzheimer’s disease. This review highlights the functions of full-length ADNP and NAP in early brain development, particularly in neurogenesis and neurite formation during cortical development. We will also discuss the potential of NAP as a therapeutic medication for neurodevelopmental disorders, especially ASD and ADNP syndrome.

## 1. Introduction

**Figure 1:**
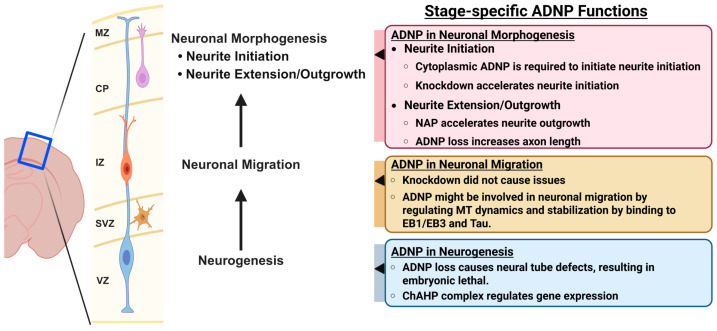
Development of the cortex: sequential cellular events and stage-specific ADNP function. Neurogenesis happens in both the ventricular and subventricular zones (VZ and SVZ). After neuronal progenitor cells exit the cell cycle, postmitotic neurons start migrating across the intermediate zone (IZ) and eventually reach the cortical plate (CP). Then, neurons start extending primitive neurites (neurite initiation), and some of them extend longer (neurite extension/outgrowth). Finally, one of them becomes an axon, and the others become dendrites. Both ADNP and NAP exhibit stage-specific functions during sequential stages of neurogenesis, migration, neurite initiation, and neurite extension. Image was created by using BioRender.com and Microsoft PowerPoint.

The development of the cerebral cortex is a highly orchestrated process involving the precise regulation of neural progenitor proliferation, differentiation, and the subsequent formation of intricate neuronal circuits (Figure 1) [1,2,3]. This complex sequence of events is governed by a multitude of intrinsic and extrinsic factors, and its disruption can lead to severe neurodevelopmental disorders [4]. Among these key regulators, the Activity-Dependent Neuroprotective Protein (ADNP) has emerged as a critical factor in brain development and function [5,6]. Its importance is underscored by the fact that haploinsufficiency of the *ADNP* gene is a leading cause of syndromic autism, known as Helsmoortel-Van der Aa syndrome, which is characterized by multiple abnormalities [7,8]. This disorder is now recognized as one of the most common single-gene causes of ASD, impacting approximately 0.17% of individuals with ASD [7,9]. Patients with ADNP syndrome exhibit a wide range of symptoms, including autistic behaviors, developmental delay without loss of developmental milestones, and mild to moderate intellectual disability (ID) [7]. Affected individuals have severe delays in both motor and speech development; the majority of patients are non-verbal [10]. The associated clinical phenotype is variable, but in more severe cases, a pattern can be seen with an array of physical and medical symptoms, including craniofacial dysmorphism (prominent forehead, high anterior hairline, thin upper lip), hypotonia, eating disorders, gastrointestinal abnormalities, as well as ocular abnormalities such as strabismus and ptosis [7]. Other common comorbid conditions include congenital heart defects, sleep disorders, and brain malformations that can be visualized on MRI scans, such as ventriculomegaly and delayed myelination [7]. An unusual and pathognomonic sign that has been reported in more than 80% of patients is the premature eruption of primary teeth [11].

At the molecular level, ADNP functions as a transcriptional regulator through chromatin remodeling complexes, such as the ChAHP (CHD4-ADNP-HP1) complex, and interacts directly with the BAF (BRG1/BRM-Associated Factor) complex, both of which are essential for modulating gene expression during development [12,13]. By binding to these complexes, ADNP orchestrates the expression of a vast network of genes crucial for organogenesis, particularly neurogenesis. Studies using *Adnp* knockout mouse models have demonstrated its essential role in neural tube closure and in the expansion of upper-layer neurons in the neocortex [6,12]. Furthermore, ADNP has been shown to promote neural differentiation by enhancing Wnt/β-catenin signaling, a pathway fundamental for embryonic development and cell fate determination [14]. The disruption of these intricate regulatory functions due to ADNP deficiency leads to impaired synaptic function, reduced dendritic spine density, and the cognitive and behavioral deficits observed in ADNP syndrome [15].

An eight-amino-acid peptide, NAP (NAPVSIPQ), also known as Davunetide, is derived from the neuroprotective domain of ADNP. NAP has been shown to promote neurite outgrowth and protect neurons from a variety of insults, suggesting its therapeutic potential for a range of neurological conditions [16]. The primary mechanism of action for NAP involves its interaction with the microtubule cytoskeleton. Microtubules are essential for neuronal structure, transport, and plasticity, and their stability is crucial for neurite formation and elongation. NAP modulates the tubulin pool, promoting microtubule dynamics and stability, which in turn facilitates these processes and supports neuronal network formation [17]. While some studies suggest an indirect mechanism of action through signaling pathways like Extracellular signal-Regulated Kinase (ERK) and Akt [18,19], recent evidence also points to NAP’s ability to penetrate the cell nucleus and interact with ADNP, potentially correcting nuclear abnormalities and directly influencing gene expression [20]. Importantly, treatment with NAP has been shown to ameliorate some of the synaptic and behavioral deficits in *Adnp*-deficient mouse models, highlighting its potential as a targeted therapy for ADNP syndrome [15].

Despite the absence of any current treatment for ADNP syndrome, management of affected patients is centered around strategies aimed at symptom alleviation strategies, such as physical and occupational therapy, as well as speech therapy [21]. The increasing understanding of ADNP’s mechanistic role, however, has paved the way for potential therapeutic approaches [22]. The most advanced of these is NAP (Davunetide, CP201) [22]. Preliminary clinical studies in *Adnp* haploinsufficient mouse models that recapitulate the features of human ADNP syndrome have shown that NAP can reverse some of the deficits associated with the disorder through a mechanism that involves stimulation of microtubule (MT) dynamics, thereby preventing tauopathy [23,24]. NAP has recently received orphan drug designation from the U.S. Food and Drug Administration, and it is currently being further developed for potential use in clinical trials for ADNP syndrome [20]. Other promising approaches that are currently being investigated, such as low-dose ketamine, have shown preliminary positive effects in small patient cohorts, although the underlying mechanism remains unclear [25]. Further investigation into the role of ADNP in neurodevelopment and disease will build on the foundations established by this study, paving the way for novel therapeutic interventions aimed at improving the lives of individuals affected by this disorder.

In summary, ADNP and its derivative peptide NAP are central players in the intricate processes of neurogenesis and neurite formation within the developing cortex. ADNP acts as a master regulator of gene expression through its involvement in chromatin remodeling, while NAP provides a more direct influence on the cytoskeletal machinery essential for neuronal morphogenesis and survival. The severe consequences of ADNP dysfunction, coupled with the promising therapeutic effects of NAP, underscore the importance of this system in brain development and its potential as a target for intervention in neurodevelopmental disorders. This review will delve deeper into the molecular mechanisms governing the functions of ADNP and NAP, exploring their interplay in shaping the developing brain and their implications for human health.

Whereas previous reviews have largely treated ADNP and NAP separately or emphasized their neuroprotective roles in the mature or aging brain, the present review focuses specifically on early cortical development. In early development, we integrate ADNP’s nuclear chromatin-remodeling functions and its cytoplasmic microtubule-regulating functions. We also link these functions mechanistically through 14-3-3/PKC-dependent nucleocytoplasmic redistribution. We further emphasize the often-overlooked distinction between neurite initiation and neurite extension, and connect these cell-biological mechanisms to the clinical features of ADNP syndrome/ASD and to NAP-based therapeutic strategies.

## 2. ADNP

**Figure 2:**
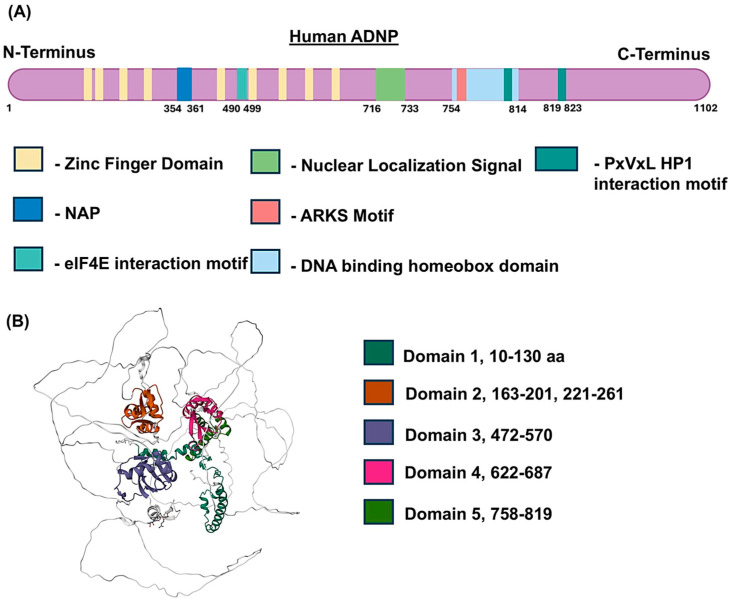
ADNP structure and functional domains. (**A**) Schematic illustration of ADNP structure. Image was created by using BioRender.com and Microsoft PowerPoint. (**B**) 3D structure of ADNP predicted by AlphaFold Version 1.

Activity-dependent neuroprotective protein (ADNP) is a vital protein for brain development, neurogenesis, and neural protection [6,13]. Initially known as a glial neuroprotection protein whose expression is regulated by vasoactive intestinal peptide (VIP), ADNP is now recognized as a multifunctional protein that is widely expressed in the body, including in the brain, heart, kidneys, skeletal muscle, and other tissues [26,27]. The human *ADNP* gene is located on chromosome 20q13.13 and encodes a 1102-amino acid protein with distinct functional motifs (Figure 2) [27,28]. The protein contains nine C2H2-type zinc fingers, a DNA-binding homeobox domain, a nuclear localization signal (NLS), and a heterochromatin protein 1 (HP1)-binding motif, all of which indicate its function as a transcription factor [28,29,30]. Additionally, ADNP has a short 8-amino-acid neuroprotective sequence called NAP (sequence: NAPVSIPQ), which has been used to develop the potential drug, Davunetide (CP201) [20,26].

The main function of ADNP is to regulate gene expression through chromatin remodeling [13]. It is a component of several chromatin remodeling complexes, such as the SWI/SNF (also known as BAF) and ChAHP complexes [13,31]. Through its interactions with core subunits such as BRG1 (Brahma-related gene 1), BAF250A, and CHD4, ADNP directs these complexes to specific regions of DNA, enabling the expression of numerous developmental and neuronal genes [13,31]. Beyond chromatin remodeling, ADNP has additionally been reported to display methyltransferase activity and to modulate DNA and histone methylation within an ADNP–WDR5 (WD Repeat Domain 5)–HDAC2 (Histone Deacetylase 2) complex [32]. ADNP also plays a role in core cellular signaling pathways. For example, ADNP also modulates the Wnt/β-catenin signaling pathway, which is described in detail in Section 3.1. A recent Adnp mouse model further links disruption of this pathway to chromatocytoskeletal defects and autism-related behavior [14,33,34]. Its interaction with cytoskeletal components such as microtubule end-binding proteins (EB1/EB3) and SHANK3 also suggests a role for ADNP in maintaining synaptic structure and function [23,35].

The NAP domain (NAPVSIPQ) contains the SIP (Ser-Ile-Pro) motif, which is a part of the SxIP motif family (Ser–any residue–Ile-Pro) [36]. The SIP motif serves as a localization signal, facilitating the binding of plus-end tracking proteins (+TIPs), such as end-binding proteins (EBs), to the plus end of microtubules (MTs) [37]. The SxIP motif binds to the EB1 homology domain (EBH), which is often identified in EBs [38]. Consistent with this, ADNP binds to EBs, as demonstrated by immunoprecipitation experiments [39]. Thus, the NAP domain is one of the most functionally important domains of ADNP and regulates microtubule dynamics by binding to EBs during neuronal development.

## 3. ADNP and NAP in Neurogenesis

The generation of new functional neurons from neural progenitor cells (NPCs) is referred to as neurogenesis. This process is critical for the normal development of the brain and continues in specific regions of the adult brain [40,41,42,43]. The formation of new neurons is controlled by multiple intrinsic and extrinsic regulators [44,45]. In this section, we discuss ADNP and NAP as regulators for neurogenesis.

### 3.1. ADNP as a Chromatin Modulator

**Figure 3:**
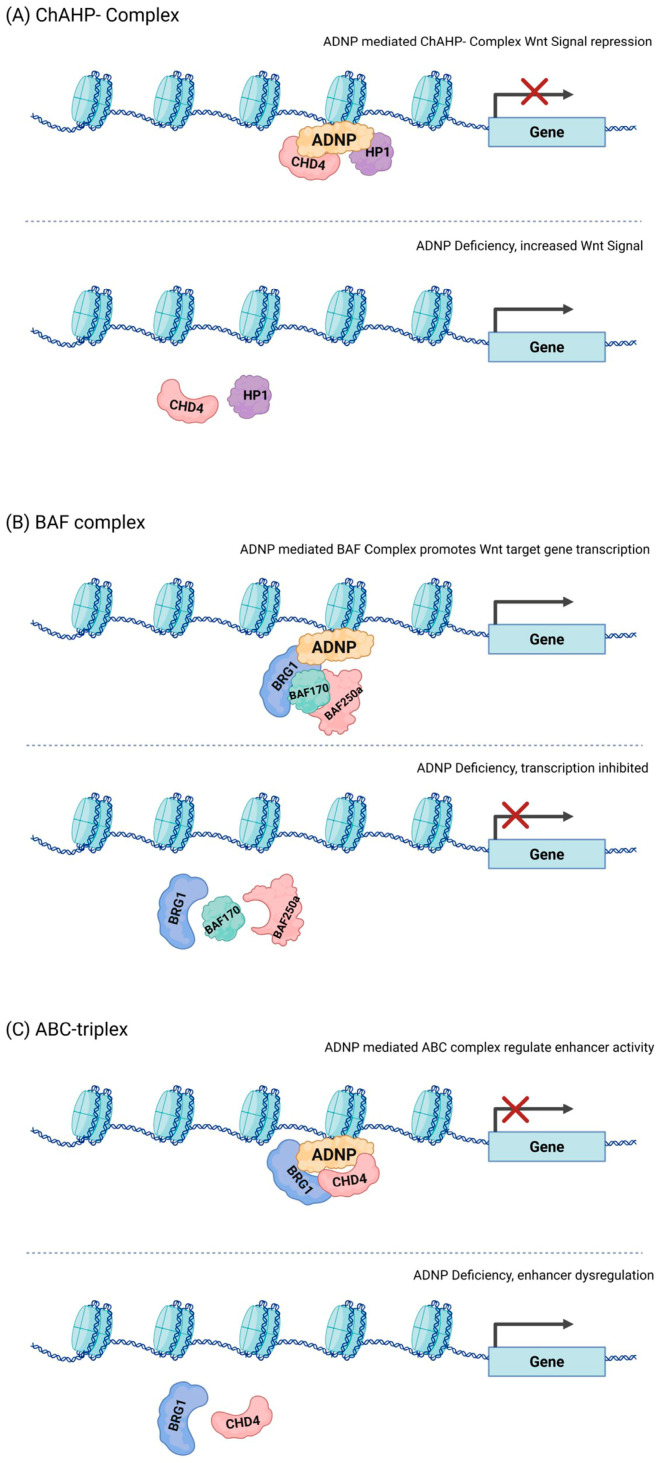
The functions of ADNP as a master chromatin regulator. (**A**) ADNP is a core subunit of the ChAHP complex (CHD4, ADNP, HP1), where it directly recruits the chromatin remodeler, CHD4 and the heterochromatin architectural protein, HP1. (**B**) ADNP binds to core components of the SWI/SNF (BAF) chromatin remodeling complex, which includes BRG1, BAF170, and BAF250a. (**C**) ADNP is also a core subunit of the ABC triplex (CHD4, ADNP, BRG1) that regulates the enhancer activity and the ratio of H3K4me3/H3K27me3 ratio. Images were created by using BioRender.com and Microsoft PowerPoint.

**Figure 4:**
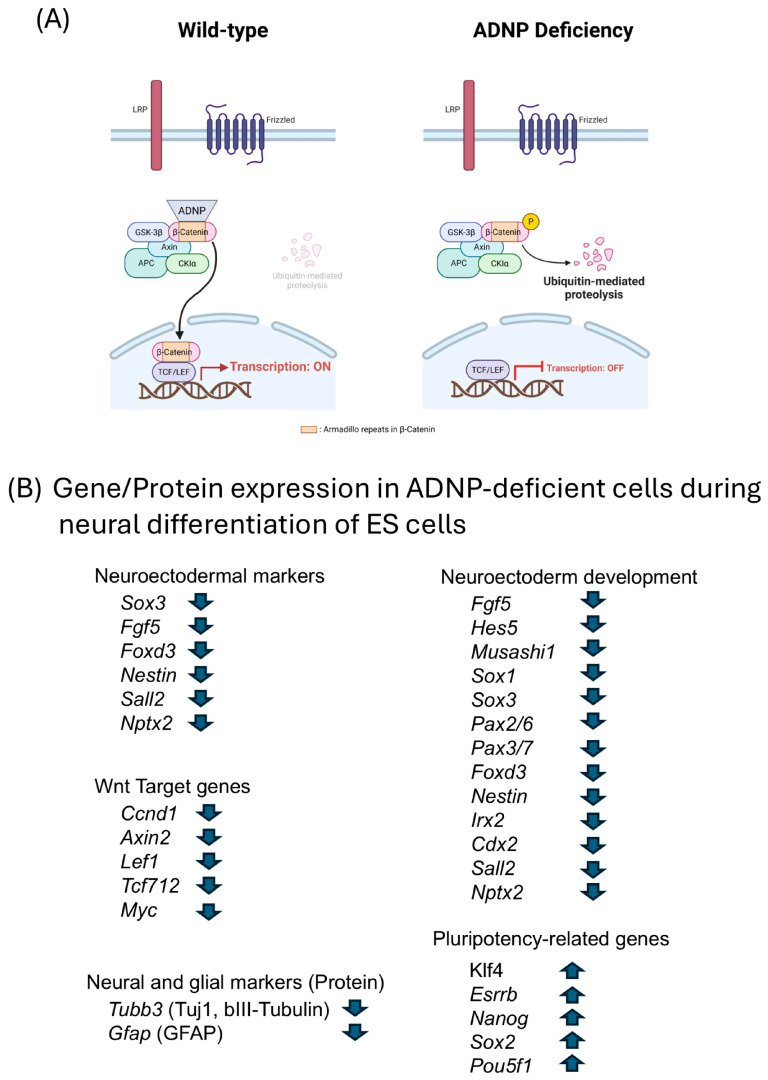
ADNP regulates neural induction and differentiation by modulating Wnt/β catenin signaling. (**A**) ADNP binds to the armadillo domain of β-catenin, stabilizing β-catenin and preventing its interaction with the ubiquitin-mediated degradation complex. This interaction between ADNP and β-catenin prevents β-catenin degradation. Thus, β-catenin regulates gene transcription. In contrast, loss of ADNP enhances formation of the β-catenin degradation complex and promotes its ubiquitin-mediated degradation, leading to reduced expression of key neuroectodermal and Wnt target genes. (**B**) ADNP deficiency affects the expression of multiple genes. These include genes involved in neuroectodermal development and associated markers. Also, the expression of Wnt-targeted genes, pluripotent-related genes, and neural and glial markers is altered by ADNP deficiency. Images were created by using BioRender.com and Microsoft PowerPoint.

The functions of NAP are linked to its parent protein, ADNP, which is essential for neurodevelopment [46]. ADNP is a component of the ChAHP complex that silences the expression of developmental genes required for embryonic development and organ growth (Figure 3) [47]. Recent studies have demonstrated that ADNP is critical for the maintenance of progenitor cell proliferation during the expansion phase of upper-layer neurons of the cortex [12]. Wnt/β-catenin signaling pathway is modulated by ADNP (Figure 4) [14,48,49]. These studies highlight the importance of ADNP in neurodevelopment, as mutations in the *ADNP* gene are the underlying cause of neurodevelopmental disorders, such as ADNP syndrome and ASD [10]. ADNP has also recently been shown to be essential for sex-dependent hippocampal neurogenesis, a function that NAP can partially compensate [50].

ADNP was initially proposed as a transcription factor based on the presence of a homeobox domain in its structure, a classic DNA-binding feature typical of many transcription factors [27]. It is now also known that ADNP plays a central role in chromatin remodeling, acts as a broad regulator of gene expression, and impacts the expression of more than 400 genes during the crucial early embryonic stages of brain development [47].

ADNP acts as a key chromatin modulator by physically interacting with BRG1 and CHD4 chromatin remodelers that control chromatin organization and gene expression [47]. ADNP binds CHD4 in a complex with the chromatin architecture protein HP1 to create a stable complex, ChAHP, that is critical for generating late-born, upper-layer neurons in the developing neocortex [31,47]. ADNP promotes neural induction and differentiation by binding to the armadillo domain of β-catenin, thereby preventing its recruitment to the degradation complex and stabilizing it; this upregulates neuroectodermal developmental genes [14]. Although the mechanisms of ADNP-mediated gene regulation are becoming clearer, its precise roles remain incompletely understood, particularly how *ADNP* mutations affect transcriptional function and whether transcriptional abnormalities are a primary cause of symptoms observed in ADNP syndrome.

ChAHP is a transcriptional regulator that regulates chromatin structure and silences short, interspersed element (SINE) B2 retrotransposons. [51]. The ChAHP protein complex acts as a local modulator of chromatin architecture by competing with the structural protein, CCCTC-binding factor (CTCF), for binding sites on the DNA. ADNP deficiency does not destroy the global 3D structure of the genome. However, it leads to significant local rewiring. The cohesin complex creates loops by extruding DNA. ADNP can act as a barrier by masking potential CTCF sites and counteracting loop formation. When ADNP is absent, CTCF attaches to these exposed sites and captures cohesin, which results in the formation of new unintended loops. These newly exposed ADNP/CTCF sites trap cohesin, making it impossible for it to reach its primary loop anchors. In turn, this can affect gene expression. Consistent with this, recent work in neural progenitor cells shows that ADNP regulates chromatin architecture and lineage fidelity through both CTCF-dependent and CTCF-independent mechanisms [52].

### 3.2. Effects of NAP on Neural Progenitor Cells via Cytoskeletal and Signaling Pathways

NAP performs its role via multiple signaling pathways by modulating the NPC function. NAP mainly affects the stability and dynamics of microtubules [17]. Microtubules are involved in numerous cellular processes, ranging from cell motility to neuronal development. NAP treatment has been shown to increase MT assembly and stability and promote neurite outgrowth [17,53]. This is also supported by the findings that NAP increases the expression of the neuronal marker, β3-tubulin [17]. However, a subsequent study reported that NAP did not bind to tubulin directly and therefore suggested an indirect effect of NAP on microtubules [19]. In addition to microtubules, it has been reported that NAP interacts with microtubule end-binding proteins, EB1 and EB3. These proteins regulate microtubule dynamics, which are responsible for axonal transport. They are also crucial for synapse development and maturation and the promotion of synaptic plasticity [22].

In addition to its effects on the cytoskeleton, NAP triggers important signaling pathways for cell survival and proliferation. Studies have demonstrated that NAP activates the mitogen-activated protein kinase/extracellular signal-regulated protein kinase (MAPK/ERK) and the phosphatidylinositol-3-kinase (PI3K)/Akt pathways [18]. Both the MAPK/ERK and PI3K/Akt pathways are well-established regulators of NPC proliferation, survival, and differentiation [54]. Through activation of these cascades, NAP creates a cellular environment that supports neuronal growth and neuronal plasticity, particularly in situations of developmental damage, such as prenatal ethanol exposure [18]. Furthermore, as a functional component of ADNP, NAP can compensate for ADNP deficiency [50]. The compensatory role suggests that NAP may indirectly help promote Wnt-mediated neurogenesis, as ADNP is a well-known stabilizer of β-catenin [14].

### 3.3. NAP in Apoptosis and Oxidative Stress

Neurogenesis is highly affected by the suppression of apoptotic cell death in both NPCs and immature neurons [55]. It has been shown that NAP has robust anti-apoptotic activity in multiple experimental models [56]. The administration of NAP markedly reduced alcohol-induced apoptosis in the primordium of the cerebral cortex and basal ganglionic eminence in a mouse model of fetal alcohol syndrome [57]. NAP also prevents alcohol-induced changes in the expression of brain-derived neurotrophic factor (BDNF) in mouse embryos [58]. This preserves a critical neurotrophic signal required for the survival and differentiation of NPCs. In neonatal hypoxia-ischemia models, NAP decreased acute cerebral oxidative stress and provided prolonged protection against brain injury and cognitive impairment [56]. Thus, NAP can protect NPCs by reducing oxidative damage and apoptosis in both normal and disease conditions in cortical neurons in culture.

In summary, NAP (Davunetide) has multiple functions that support neural progenitor cell proliferation and neurogenesis. NAP provides the cellular and molecular conditions necessary for successful neurogenesis by suppressing apoptosis and attenuating oxidative stress. Since NAP is effective at femtomolar concentrations, it could have potential as a therapeutic agent for neurodevelopmental and neurodegenerative conditions where neurogenesis is impaired.

## 4. Intracellular ADNP Distribution (Nucleus vs. Cytoplasm) Throughout Neuronal Differentiation

**Figure 5:**
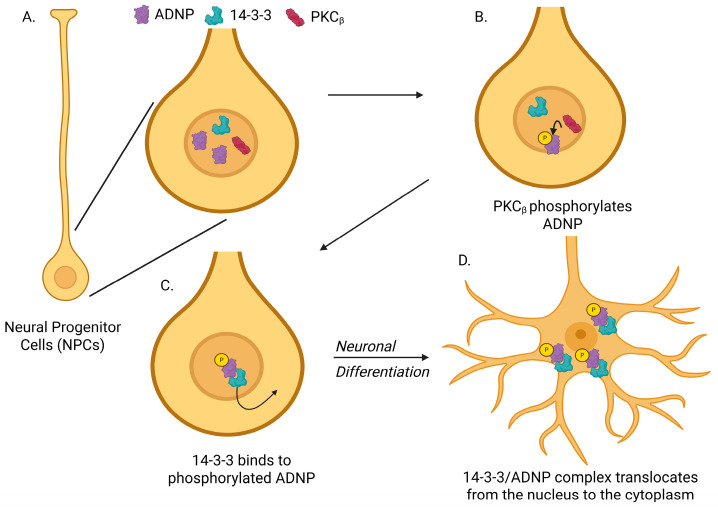
Schematic illustration of the regulation of mouse ADNP distribution by 14-3-3 and PKC. In neuronal progenitor cells (NPCs), Adnp, 14-3-3, and PKCβ are predominantly localized in the nucleus (**A**). As neurons differentiate, PKCβ phosphorylates ADNP (**B**), which facilitates the interaction between ADNP and 14-3-3 (**C**). Then, the ADNP/14-3-3 complex translocates from the nucleus to the cytoplasm during neuronal differentiation (**D**). Image was created by using BioRender.com and Microsoft PowerPoint.

The subcellular distribution of ADNP appears to be a key determinant of its distinct functions during neuronal differentiation. Mandel et al. investigated how ADNP expression and localization relate to cell fate and neurite outgrowth. By differentiating pluripotent P19 cells into endodermal and neuronal lineages, the authors demonstrated that ADNP was distributed in the nucleus of pluripotent and endodermal cells, but was enriched in neurites following neurodifferentiation [59]. This suggests that the functions of ADNP are regulated by its subcellular location during neuronal development. A recent study showed that 14-3-3 proteins control the subcellular distribution of ADNP in the cytoplasm (Figure 5) [5]. In neurospheres prepared from embryonic cortices, ADNP is predominantly distributed in the nucleus. However, when they differentiated into mature neurons, the distribution of ADNP changed, and it was predominantly distributed in the cytoplasm. Post-translational modification analysis by proteomics suggested that ADNP was phosphorylated by PKC. In silico analysis identified multiple putative 14-3-3 binding sites in ADNP, multiple phosphorylation sites, and candidate kinases, including PKC. Based on these analyses, serine 42 on ADNP is a potential PKC phosphorylation site. Inhibitor-based experiments showed that PKC inhibitor treatment in mature cortical neurons retained ADNP in the nucleus and disrupted normal neurite formation. These findings suggest that in neuronal progenitor cells, ADNP is predominantly localized in the nucleus and regulates gene expression through chromatin-associated mechanisms, whereas during neuronal differentiation and maturation, ADNP is phosphorylated by PKC, interacts with 14-3-3 proteins, and is shuttled to and maintained in the cytoplasm, where it regulates MT dynamics. This compartment-specific model serves as a valuable framework for understanding how ADNP links developmental gene regulation to later cytoskeletal remodeling during neuronal maturation. Recent clinical findings further link ADNP frameshift variants to nucleocytoskeletal alterations in patients, supporting the relevance of this compartment-specific model to ADNP syndrome [60].

## 5. ADNP as a Regulator of Neurite Formation

### 5.1. ADNP Regulates Neurite Outgrowth

Following its developmental relocation to the cytoplasm, ADNP appears to play an important role in neurite formation and neuronal morphogenesis. After neurogenesis and neuronal migration are completed during cortical development, neurite formation begins at the final destination of pyramidal neurons in the cortex (Figure 1) [61]. In mice, pyramidal neurons in layer 2/3 labeled by in utero electroporation (IUE) at E15.5 complete neuronal migration and reach the upper site of the cortical plate by P0, and then initiate neurite formation [5]. Before becoming an axon or dendrite, a neuronal branch is known as a neurite. Neurons start creating protrusions where F-actin accumulates. MTs invade these protrusions, thus extending and stabilizing them [62]. During development, the localization of ADNP changes from a nuclear to a cytoplasmic distribution concurrently with neurite formation [5]. A recent study suggests that the movement of ADNP to the cytoplasm is controlled by 14-3-3 proteins during neurite formation in primary cortical neurons [5].

The functions of ADNP in neurite formation have been examined in both *in vitro* and *in vivo* cortical neuron models [5]. When ADNP was knocked down in primary cortical neurons using shRNA, the number of neurites increased, and the longest neurite (which would likely become an axon) grew significantly longer than in control neurons expressing scramble shRNA. The lengths of the shorter neurites (which would likely become dendrites) were not significantly affected. These findings suggest that an ADNP may have a preferential role in axonal outgrowth. Using IUE at E15.5, ADNP was knocked down in pyramidal neurons in layer 2/3. By P15, ADNP-deficient neurons showed a significant increase in the number of basal dendrites, but these dendrites were abnormally short. These findings suggest that ADNP negatively regulates neurite initiation but positively controls neurite extension *in vivo*. There were also some observed differences regarding axonal outgrowth; axons successfully crossed the midline of the brain in normal numbers based on the thickness of axon bundles, but ADNP-deficient axons grew significantly longer once they reached the opposing hemisphere, where dense hyperinnervation was observed throughout the contralateral cortex. These observations suggest that ADNP may negatively regulate axonal outgrowth. Using time-lapse live imaging on brain slices in combination with IUE at E15.5, ADNP-deficient neurons reached the cortical plate at P0 and showed severe defects in neurite growth dynamics. Despite the rapid extension and retraction of multiple processes (primitive neurites/protrusions) by control neurons, ADNP-deficient neurons consistently grew a single primitive neurite with a significantly higher stabilization rate and a significantly lower retraction frequency. Significant swelling of neurites was also observed in ADNP-deficient neurons. These data indicate that ADNP plays a key role in neurite initiation. Consistent with a role in cortical neuron development, a recurrent *ADNP* missense variant (p.C687R) has recently been shown to disrupt chromatin regulation, neuronal migration, and dendritic arborization in mouse cortex and patient-derived neurons [63].

### 5.2. Actin Regulation by ADNP

The regulation of actin dynamics is a potential mechanism for ADNP and NAP to regulate neurite formation, particularly neurite initiation [64,65,66,67,68]. While ADNP primarily works as an MT regulator, a recent study has shown that it binds to actin [35]. This study reveals a mechanistic link between two major autism genes, ADNP and SHANK3. They directly bind to each other through the SH3 domain on ADNP. They also bind actin via the actin-binding motif of SHANK3. NAP enhances the formation of the SHANK3/ADNP/actin complex, and chronic intranasal treatment with NAP improves behavioral abnormalities, including anxiety/depression-like behavior, overgrooming, and social recognition defects in Shank3 mutant mice. ADNP also affects the localization and activity of proteins associated with membrane protrusion and cytoskeletal dynamics. The Fes/CIP4 Homolog Bin/Amphiphysin/Rvs (F-BAR) protein family, which controls membrane curvature and actin dynamics during neurite emergence, may be altered by gene expression that is controlled by ADNP [69]. Although studies on ADNP in actin regulation are limited, it would be beneficial to further investigate the role of ADNP in actin regulation in neurite formation, especially in neurite initiation.

### 5.3. ADNP Associates with EB Protein (MT Plus-End Binding Proteins)

Besides actin regulation, ADNP regulation of MT dynamics might be crucial for the transition from neurite initiation to neurite extension/outgrowth [70]. To form a neurite, MTs must be stabilized, which provides both structural support for neurites and a track for membrane trafficking. The interaction of ADNP with EBs and its effects on microtubule stability are likely to determine the location and time of neurite formation [23,39].

Previous studies have shown that ADNP and its peptide NAP interact directly with EB3 proteins in a neuroblastoma model [39]. In this mechanism, ADNP binds the +TIP end-binding protein EB3 via its SxIP motif, while depletion of EB1 or EB3 abrogates cell protection. ADNP has also been found to interact with microtubule-associated protein (MAP) 1 light chain 3 (LC3), an autophagy-related protein [71]. SxIP has been proven to exhibit MT-stabilizing effects by blocking Tau dissociation and MT disassembly caused by zinc intoxication [72]. Supporting its role as an MT regulator, shRNA-mediated knockdown of ADNP decreases MT organization, resulting in altered cell morphology, including changes in cellular processes and cell number [13]. An additional study from Dr. Gozes’ lab examined NAP’s capability to affect MT polymerization and dynamics enriched with unfractionated brain MAPs and purified Tau isoforms 3R and 4R (3R for neurodevelopment and 4R for neurodegeneration) *in vitro*. It was reported that NAP did not have any effect on either assembly into microtubules or *in vitro* dynamics [19]. They did find that NAP preferentially bound to Tau 3R compared to Tau 4R in an affinity-chromatography assay using rat brain extracts [73].

Taken together, these findings support the view that ADNP contributes to neurite initiation, outgrowth, and cytoskeletal organization during cortical development.

## 6. NAP as a Regulator of Neurite Formation

### 6.1. NAP in Neurite Formation

Despite the importance of ADNP in brain formation, NAP plays a key role as an effector domain, promoting neuronal differentiation and neurite outgrowth through multiple molecular mechanisms [74]. The primary mechanism of action is the modulation of the neuronal cytoskeleton, specifically the MT network, which is crucial for the formation, extension, and maintenance of neurites [75]. NAP has been extensively studied and shown to promote neurite outgrowth in many neuronal cell types, including hippocampal, cortical, cerebellar, and retinal ganglion cells [18,53,74].

*In vitro* research has demonstrated that NAP induces strong neurite extension in rat hippocampal and cortical cultures, regions critical for learning and memory [74]. NAP also increases the survival and outgrowth of retinal ganglion cells, suggesting its therapeutic potential in optic nerve diseases caused by axonal damage [53]. The broad activity profile suggests that NAP interacts with conserved molecular machinery regulating neuronal development. NAP promotes the structural and functional maturation of different neuronal populations, leading to the formation of complex neural circuits.

NAP exerts neurotrophic effects by activating key intracellular signaling cascades involved in neuronal growth and differentiation. Studies have shown that NAP treatment stimulates MAPK/ERK and PI3K/Akt pathways [18]. These pathways are crucial in the regulation of gene expression and protein synthesis necessary for cell survival, differentiation, and plasticity. Activation of these pathways results in the downstream phosphorylation of transcription factors and other effector proteins that coordinate neurite elongation and branching. This signaling mechanism allows NAP to not only promote growth under normal developmental conditions but also protect neurons and restore growth when exposed to neurotoxic insults, such as ethanol exposure during development [18,76].

The effects of two peptides, activity-dependent neurotrophic factor-9 (ADNF-9; SALLRSIPA) and NAP, on neurite outgrowth were investigated in cultured rat hippocampal and cortical neurons [74]. These peptides are derived from or related to glial-derived activity-dependent neurotrophic factor (ADNF) and ADNP, respectively, and are known for their potent neuroprotective effects at femtomolar concentrations. ADNF-9 and NAP were found to promote neurite outgrowth in a concentration-dependent manner in hippocampal neuron culture, and their activity was maximal at femtomolar levels, as shown by immunofluorescence labeling with MAP2-FITC. Both peptides affected neurite outgrowth in hippocampal cultures, but the effect was less noticeable in cortical cultures.

Because NAP exerts its principal activity intracellularly, an important question concerns how this peptide reaches its cytoplasmic and nuclear targets. It remains active at femtomolar concentrations and is effective after intranasal and systemic administration, consistent with efficient cellular uptake and penetration of the central nervous system [16,46,77]. To date, no classical high-affinity cell-surface receptor for NAP has been identified. The previous study suggested that NAP can enter cells by endocytosis, which is assisted by dynamin [78]. Thus, current evidence indicates that NAP enters cells by dynamin-assisted endocytosis rather than receptor-mediated endocytosis.

### 6.2. Microtubule Regulation by NAP

The interaction between NAP and tubulin, the protein subunits of MTs, has been proposed as a key mechanism underlying neurite outgrowth. The structural integrity and dynamic extension of axons and dendrites are critical processes supported by NAP through the promotion of MT assembly and stabilization [46,79]. NAP treatment has been found to significantly affect the tubulin pool in neurons, influencing the tyrosination cycle of α-tubulin and increasing the overall MT network area. Through this modulation, the cytoskeleton becomes more robust and dynamic, enabling it to support the advancement of the growth cone, the motile structure at the tip of a growing neurite [17]. Although NAP clearly stabilizes MTs in living neurons, research using isolated tubulin in cell-free systems has shown that it does not directly affect the velocity of polymerization or behavior of reconstituted MTs. This implies that the effects of NAP are mediated through indirect pathways that rely on cell signaling, rather than direct binding to tubulin [17]. These indirect pathways enhance MT stability and prevent cytoskeletal collapse. NAP can restore neuronal dysfunction in tauopathy models by compensating for the loss of Tau’s stabilizing function, which underscores its critical role in maintaining cytoskeletal integrity [80,81].

The impact of NAP on MT dynamics is enhanced by its interaction with microtubule end-binding proteins (EBs), specifically EB1 and EB3. The interaction between NAP and EBs is mediated by the Ser-Ile-Pro (SIP) motif in NAP [82]. These proteins are found at the plus-ends of developing MTs and play a key role in regulating MT dynamics. By interacting with EB1 and EB3, NAP maintains the directional development of MTs in the growth cone periphery [15,71]. This targeted action enables the physical extension of the neurite in the extracellular environment. NAP, EBs, and Tau interact to create a synergistic effect that significantly augments Tau-MT associations [25]. The NAP–EB1–Tau microtubule-stabilizing mechanism has also been demonstrated in non-neuronal epithelial cells, underscoring its general relevance [83].

Through its direct and indirect interactions with the MT cytoskeleton, NAP promotes neurite formation [84]. It has been demonstrated that NAP can cause MT reorganization in living cells, which provides a structural basis for neurite extension [75]. The tubulin tyrosination cycle is a post-translational modification that reflects MT stability [17]. NAP treatment results in changes in MT network strength and plasticity by increasing the levels of both stable, long-lived detyrosinated microtubules (Glu-MTs) and newly formed, dynamic tyrosinated microtubules (Tyr-MTs) [17]. The total polymerized α-tubulin network area within neuronal-like PC12 cells after NAP administration increases in a concentration-dependent manner [17].

### 6.3. NAP in MT Homeostasis/Axonal Transport

Recent research has established that NAP stabilizes MTs by interacting with proteins like Tau and proteins that bind to the MT ends [85]. It is especially important to consider the modulation of Tau polymerization because Tau hyperphosphorylation and the consequent polymerization are characteristic hallmarks of various neurodegenerative diseases such as Alzheimer’s disease (AD) [72]. By inducing Tau association with MTs, this cross-talk prevents the generation of neurofibrillary tangles and exerts neuroprotective effects [23].

Furthermore, studies have shown that the structural features of NAPs are similar to a neuroprotective subregion of Tau and tubulin, implying a potential shared pathway for MT stabilization [86]. The NAP motif is involved in MT dynamics and is implicated in the stabilization of the dendritic column, which is essential for both synaptic plasticity and memory consolidation [82]. NAP is also important for proper axonal transport, a key component of neuronal health [79]. Disruption of axonal transport pathways is a key issue in neurodegenerative disease pathology, causing synapse damage and neuronal cell death [87]. The effects of NAP treatment on axonal transport are supported by its action against external insults, including excitotoxicity, which is frequently involved in AD and other tauopathies [76]. The association between MT stabilization and neuroprotection suggests that interventions targeted to this axis might provide therapeutic strategies to reverse or mitigate the progression of neurodegenerative disorders.

Furthermore, this study proposes a model in which MT-stabilizing peptides exert neuroprotective effects by modulating Tau pathology [88]. This strategy is consistent with current therapeutic approaches targeting Tau signaling and axonal transport. At the molecular level, Ghosh and Singh have highlighted that dysregulated MTs and cargo transport play a crucial role in the onset of neurodegeneration [89].

In summary, NAP’s multifaceted role in MT regulation strongly supports its potential use in therapeutic strategies for neurodegenerative diseases. Although NAP cannot reverse aging or eliminate cognitive decline, it is a promising candidate for neurotherapy, warranting further investigation. Future studies will be essential to establish viable clinical applications and further elucidate the mechanism underlying NAP’s neuroprotective effects. Although further work is needed to define its developmental specificity and clinical relevance, current evidence supports NAP as both a mechanistically informative effector of ADNP signaling and a potential therapeutic.

## 7. ADNP Syndrome/ASD and Its Intervention

### 7.1. ADNP Syndrome/ASD

The disruption of ADNP function is directly clinically relevant, as pathogenic variants in *ADNP* lead to a syndromic form of autism spectrum disorder that is characterized by multisystem neurodevelopmental abnormalities. *ADNP* has been identified as an ASD risk gene [90,91,92]. Mutations in *ADNP*, located on chromosome 20q13, are usually heterozygous and lead to ADNP syndrome (OMIM # 615873), a neurodevelopmental disease primarily defined by autism, intellectual disability, and dysmorphic facial features. The prevalence of *ADNP* mutations in cases with ASD is 0.17% [9].

The clinical phenotype of ADNP syndrome was described within the last 10 years. ADNP syndrome, also referred to as Helsmoortel-Van der Aa syndrome (HVDAS), was first described in patients in 2014 [9,93]. *ADNP* was also deemed a novel developmental disorder gene by the Deciphering Developmental Disorders Study. In the global cohort (n = 78; 44 males and 34 females), the most frequently reported clinical characteristics included ID (100.0%), speech delay (98.6%), motor delay (95.9%), and ASD features (92.8%) [7]. Additional neurological features included hypotonia (78.3%), visual problems (73.6%), sleep problems (65.2%), hand and foot abnormalities (62.3%), structural brain abnormalities (55.9%), attention-deficit/hyperactivity disorder (43.9%), congenital heart disease (37.7%), and seizures (16.2%).

This disorder is characterized by defects in chromatin modification caused by *ADNP* mutations [94]. ADNP functions in multiple chromatin remodeling complexes, including the SWI/SNF complex, ChAHP complex, and ADNP-BRG1-CHD4 complex (Figure 3) [29]. ADNP deficiency leads to dysregulation of chromatin remodeling within these complexes, resulting in transcriptional alterations.

Numerous genotype-phenotype correlations have been observed in ADNP syndrome patients. 25 nonsense and 21 frameshift mutations were reported in 78 ADNP syndrome patients. The p.Tyr719* mutation was one of the mutation hotspots and was associated with a delayed age of walking and sensory processing issues, such as insensitivity to pain, compared to other variants [7]. Variants between RNA nucleotides 2000–2340, including the p.Tyr719* mutation, were also associated with increased age at walking and a higher rate and severity of ASD [95].

The pathogenic mechanism of autism in ADNP syndrome, caused by mutations in the *ADNP* gene, has been elucidated during the past few years. NAP and ketamine are also being highlighted as candidate drugs for ADNP syndrome [29]. Recently, D’Incal et al. described a patient with ADNP syndrome with a novel *ADNP* mutation resulting in haploinsufficiency [96]. Since NAP treatment partially rescued cognitive deficits in haploinsufficient mouse models, NAP is a potential therapeutic option for this kind of mutation [15]. Additionally, an open-label study comprising ten ADNP syndrome patients demonstrated the safety and efficacy of low-dose ketamine, possibly through upregulation of ADNP expression [25].

### 7.2. Therapeutic Strategies for ADNP Syndrome

There is currently no approved disease-modifying treatment for ADNP syndrome, and management remains supportive (physical, occupational, and speech therapy) [21]. The most advanced candidate is NAP (davunetide, CP201), the eight-amino-acid microtubule-stabilizing fragment of ADNP. In Adnp-haploinsufficient mouse models that recapitulate features of human ADNP syndrome, NAP rescues synaptic, behavioral, and Tau-related deficits through stimulation of microtubule dynamics and protection against tauopathy [15,23,24]. NAP is amenable to non-invasive intranasal delivery and crosses the blood–brain barrier, with further clinical development ongoing [22,77]. Clinical evaluation of systemic (intravenous) davunetide has additionally revealed sex-dependent differences in drug metabolism and neuroprotection [97]. A second strategy is low-dose ketamine. An open-label study in ten children with ADNP syndrome reported a favorable safety profile and behavioral and electrophysiological improvements, possibly mediated by upregulation of ADNP expression [25,98]. Emerging preclinical strategies that target the downstream consequences of ADNP deficiency include inhibition of the histone demethylase Lysine-Specific Demethylase 1 (LSD1), which ameliorates synaptic deficits in Adnp-mutant mice, and mechanistic Target of Rapamycin (mTOR) inhibition with everolimus, which improves cognitive and synaptic dysfunction following prefrontal ADNP knockdown [99,100]. Together, these efforts illustrate how an improving mechanistic understanding of ADNP and NAP is beginning to translate into rational and targeted therapeutic strategies. However, controlled clinical trials are still required to establish efficacy.

Taken together, these findings indicate that ADNP syndrome can be used as a clinically informative framework to link developmental dysregulation of chromatin remodeling and neuronal morphogenesis to autism-related phenotypes.

## 8. Conclusions and Future Directions

**Summary.** ADNP is a multifunctional regulator of early brain development that links transcriptional control and cytoskeletal organization. In the nucleus, it acts within chromatin-remodeling complexes (ChAHP, SWI/SNF) to govern developmental gene expression, whereas in the cytoplasm, following 14-3-3/PKC-dependent redistribution, it regulates microtubule dynamics to control neurogenesis and neurite formation.

**Open questions.** It remains unclear how specific *ADNP* mutations differentially affect its nuclear versus cytoplasmic functions, how the timing of nucleocytoplasmic shuttling is controlled, and whether transcriptional or cytoskeletal dysregulation is the primary cause of ADNP syndrome phenotypes. The relationship between ADNP/NAP and actin-based mechanisms of neurite initiation also warrants further study.

**Future directions.** Combined analysis of Adnp knockout mouse models and neurons derived from patient-specific human induced pluripotent stem cells (hiPSCs), together with high-resolution live imaging and stage-specific manipulation, will be needed to dissect these mechanisms. These systems, integrated with translational and pharmacological approaches, will help clarify ADNP’s role across neurodevelopment and accelerate the evaluation of NAP and other ADNP pathway-targeted therapies.

## Data Availability

No new data were created or analyzed in this study. Data sharing is not applicable to this article.

## Abbreviations

The following abbreviations are used in this manuscript:

ADNF	Activity-dependent neurotrophic factor
ADNP	Activity-dependent neuroprotective protein
ASD	Autism spectrum disorder
BAF	BRG1/BRM-associated factor
BDNF	Brain-derived neurotrophic factor
ChAHP	CHD4-ADNP-HP1 complex
CTCF	CCCTC-binding factor
EB	End-binding protein
EBH	EB homology domain
ERK	Extracellular signal-regulated kinase
HVDAS	Helsmoortel-Van der Aa syndrome
HP1	Heterochromatin protein 1
ID	Intellectual disability
IUE	In utero electroporation
LC3	Microtubule-associated protein 1 light chain 3
MAP	Microtubule-associated protein
MAPK	Mitogen-activated protein kinase
MRI	Magnetic resonance imaging
MT	Microtubule
NAP	NAPVSIPQ
NLS	Nuclear localization signal
NPC	Neural progenitor cell
PI3K	Phosphatidylinositol 3-kinase
PKC	Protein kinase C
SH3	Src homology 3
SINE	Short interspersed nuclear element
SIP	Ser-Ile-Pro
SWI/SNF	Switch/sucrose non-fermentable
VIP	Vasoactive intestinal peptide
